# Serious and persistent suicidality among European sexual minority youth

**DOI:** 10.1371/journal.pone.0240840

**Published:** 2020-10-16

**Authors:** Pietro Gambadauro, Vladimir Carli, Danuta Wasserman, Judit Balazs, Marco Sarchiapone, Gergö Hadlaczky

**Affiliations:** 1 National Centre for Suicide Research and Prevention of Mental Ill-Health (NASP), Department of Learning, Informatics, Management and Ethics (LIME), Karolinska Institutet, Stockholm, Sweden; 2 Department of Women’s and Children’s Health, Uppsala University, Uppsala, Sweden; 3 Res Medica Sweden, Uppsala, Sweden; 4 Eötvös Loránd University, Institute of Psychology, Budapest, Hungary; 5 Bjørknes University College, Oslo, Norway; 6 Department of Medicine and Health Science, University of Molise, Campobasso, Italy; Universita Cattolica del Sacro Cuore Sede di Roma, ITALY

## Abstract

**Background:**

Suicide is a leading cause of death among adolescents and more knowledge from high risk groups is needed in order to develop effective preventive strategies. The aim of this study was to evaluate the association between sexual minority status and suicidality in a multinational sample of European school pupils.

**Methods:**

A self-report questionnaire was delivered to 2046 adolescents (mean age 15.34±1.01; 56.3% females) recruited from 27 randomly selected schools in 6 European countries. Suicidal ideation, measured with the Paykel Suicide Scale (PSS), and lifetime suicide attempts were compared between heterosexual and sexual minority (i.e. those with a non-heterosexual orientation) youth. Poisson regression analyses studied the longitudinal association between sexual minority status and the rate of serious suicidal ideation, measured at three time-points during a 4-month period. Several variables, including alcohol and illegal drugs use, bullying, family interaction, school-related stress, economic status, and religiosity, were included in multivariable analysis. Sex-stratified analyses evaluated the association respectively among females and males.

**Results:**

Of 1958 pupils included in analysis (mean age 15.35±1.00; females 56.8%), 214 (10.9%) were categorized as sexual minority youth (SMY). When compared to heterosexual youth (HSY), SMY were significantly more exposed to substance abuse, bullying, school-related stress, and lower economic status. SMY pupils had significantly higher suicidal ideation scores (p<0.001; r 0.145) as well as higher prevalence of serious suicidal ideation (odds ratio [OR] 2.64, 95% confidence interval [CI] 1.83–3.79) and previous suicide attempts (OR 2.72, 95%CI 1.77–4.18), compared to their HSY peers. The rate of serious suicidal ideation reports during the study was significantly higher among SMY compared to HSY (rate ratio [RR] 2.55, 95%CI 1.90–3.43). A significant difference was found even when controlling for the pupils’ country as well as after adjustment for alcohol and illegal drugs use, bullying, family interaction, school-related stress, economic status, and religiosity (adjusted RR 1.73, 95%CI 1.23–2.48). Stratified analyses showed significant associations between SMY status and persistent serious suicidal ideation for both sexes, with a notably strong association among male pupils (females aRR 1.51, 95%CI 1.01–2.24; males aRR 3.84, 95%CI 1.94–7.59).

**Conclusions:**

European sexual minority youth are a high-risk group for suicidality, independently from objective factors such as victimization or substance abuse. There is a need to develop primary and secondary preventive measures for sexual minority youth, including the management of context vulnerabilities and related distal stressors, before the establishment of proximal stressors. Context-targeting interventions may effectively focus on social and economic factors, as well as on the potentially different risk profile of female and male sexual minority youth.

## Introduction

Adolescent mental wellbeing is a global topic of public health concern [1]. Among contemporary youth, psychiatric symptoms are highly prevalent and suicide is a leading cause of death [1–3]. Preventive efforts targeting high risk groups are therefore urgently advocated [1].

Adolescents’ vulnerability is closely related to the intense biopsychosocial changes occurring during the transition to adulthood, including those related to their sexual development such as identity exploration and initiation [4,5]. Sexual minority youth (i.e. those “identifying as lesbian, gay, bisexual, or another non-heterosexual identity” [6]) are a recognized risk group for psychiatric symptoms and suicidal behavior [7–9]. The excess of mental health risk among sexual minority youth may be explained through a minority stress model: because of stigma, adolescents belonging to a sexual minority are exposed to social stressors (e.g. victimization, discrimination and rejection) which eventually lead to poor health outcomes and risk behaviors [10]. It may be argued that context vulnerabilities leading to social stressors are in general related to adolescent mental health, regardless of sexual orientation. However, as the exposure to minority stressors is chronic [10], it can be assumed that SMY eventually develop more persistent symptoms than their heterosexual counterparts.

Despite the chronic and context-related nature of minority stressors, the current knowledge base largely relies on data from cross-sectional surveillance surveys from North America [7,9], whereas other macro regions such as Europe are covered by individual studies at national level but suffer from a lack of coordinated data-collection efforts. Further knowledge gaps exist regarding the potential differences in suicidality between female and male sexual minority youth. Sex is indeed an epidemiological correlate of mental health as well as a plausible determinant of social interaction patterns. However, relatively few studies on suicidality and sexual orientation evaluate differences between males and females [11], the majority only reporting aggregated results [7,9].

The aim of this longitudinal study was to evaluate the association between sexual minority status and suicidality in a multinational sample of European school pupils. Specific objectives were to evaluate whether sexual minority status is an independent predictor of serious and persistent suicidal ideation, and if the predictive value varies between female and male adolescents.

## Methods

### Study population

The study population consisted of 2046 adolescents (mean age 15.34±1.01; 56.3% females) who participated in the longitudinal SUPREME (Suicide Prevention through Internet and Media Based Mental Health Promotion) project between 2012 and 2013 [12,13]. These pupils were recruited from 27 randomly selected schools in six European countries, namely Estonia (West Viru County), Hungary (Budapest), Italy (Molise), Spain (Barcelona), Sweden (Stockholm County), and the United Kingdom (eastern England). A national study center was established in each country, whereas the project was coordinated by the National Centre for Suicide Research and Prevention of Mental Ill-Health (NASP), Karolinska Institutet, Stockholm. A structured self-report questionnaire was delivered to all participating pupils at baseline, in order to obtain data about socio-demographics, lifestyle, behaviors and mental health (T1). Follow-up questionnaires were delivered 2 and 4 months after the baseline (respectively, T2 and T3). During the study, the participants were allocated to three different groups in order to evaluate a mental-health promoting website [12]. Further details about SUPREME’s sample and procedures are available in previous publications [12,13]. All the pupils participating in SUPREME with available age and sex, and who responded to baseline questions about their sexual orientation and serious suicidal ideation, were included in this study. Written consent was given by all participants and, depending on local regulations, by one or two caregivers. Ethical approval was obtained from the local ethical committees of each study center.

### Measurements and variables

The exposure variable sexual orientation was investigated through a single question (“what is your sexual orientation”; possible answers including heterosexual, homosexual, bisexual, other, I don’t know). A dichotomous variable was computed by means of categorizing all answers other than heterosexual as sexual minority, as previously described [14,15].

The outcome variable suicide ideation was measured by means of the Paykel Suicide Scale (PSS) [16], at three different timepoints during the study (T1, T2 and T3). The PSS evaluates suicidal thoughts and planning occurring during the two latest weeks through four items (1. Have you felt, during the past two weeks, that life was not worth living? 2. Have you wished, during the past two weeks, that you were dead?–for instance, that you could go to sleep and not wake up? 3. During the past two weeks, have you thought of taking your life, even if you would not really do it? 4. During the past two weeks, have you reached the point where you seriously considered taking your life or perhaps made plans how you would go about doing it?), with possible answers ranging from 1 (“never”) to 7 (“always”). A suicide ideation score (or PSS score) was obtained for this study by averaging the scores of the PSS items [13], allowing for not more than one missing answer. The categorical outcome variable severe suicide ideation was defined by an answer different than “never” to the 4th item of the PSS. This definition includes all respondents who have seriously considered or planned suicide during the previous two weeks, in agreement with previous studies [3,17]. In order to define persistent suicidal ideation, a count data variable was computed by means of adding up the counts of severe suicidal ideation reports at the three timepoints, with possible values ranging between zero (i.e. outcome never reported) and three (i.e. outcome reported at all timepoints). Because an increasing count identified subjects who reported the symptom a greater number of times during the study, the resulting variable was conceptualized as a measure of persistent ideation. Previous suicide attempts, captured through a multiple-choice question (“Have you ever tried to take your own life?” with alternative choices: yes, during the past 2 weeks; yes, during the past 6 months or longer; no, never), were recoded into a dichotomous variable (yes or no).

Several additional variables were obtained thought the SUPREME questionnaire. Sex was self-reported, whereas age was calculated from the date of birth. Specific items in SUPREME’s questionnaire investigated the frequency of alcohol and illegal drugs consumption. For this study, a single dichotomous variable was computed where “substance abuse” was defined by alcohol consumption ≥ once/week and/or illegal drug use ≥ once/month. The exposure to bullying victimization was addressed by a 7-point question about being bullied (1 = very rarely or never, to 7 = very often), and a dichotomous variable was created by means of categorizing answers of 2 or above as being bullied. Worries and stress related to school activities were assessed through two separate 7-point questions with possible answers ranging from 1-very little or not at all to 7-very much. Answers of 5 or above in any of those questions were defined as school-related stress in a computed dichotomous variable. The pupils’ interaction with their family context was evaluated with a question asking how they got along with the people they live with (parents or others) on a 7-point scale, with possible answers rating from 1-very bad to 7-very good. These measurements were recoded into a dichotomous variable where a suboptimal family interaction defined as a score of 4/7 or less. The respondents’ perceived economic status was evaluated by a direct question (“how is your economic situation”) with four possible answers ranging from not being able to “buy any of the things that I really want" to having “enough money to buy most of the things that I really want”. Pupils were categorized as “lower economic status” if they could not afford any or many of the things they wished. Religiosity was evaluated with a 7-point question (“how religious are you”: not at all = 1; very religious = 7), and a dichotomous variable was computed by means of categorizing responses of 5 and above as “very religious”. An additional dichotomous variable was computed in order to define pupils with concordant or discordant religiosity when compared to their context. Discordant religiosity was defined as either religious pupil (i.e. an answer different than “not at all” at the religiosity item) in a context where the majority of their national peers is not, or vice-versa.

### Statistical analysis

The study variables were summarized through descriptive statistics. Baseline differences between heterosexual and sexual minority pupils were examined by means of chi-square test (for categorical variables) or Mann-Whitney U test (for continuous variables). Differences in rates of serious suicidal ideation between the two groups were evaluated by means of chi-square test at the three study points. A Poisson regression of counts was used to study the longitudinal association between the rate of serious suicidal ideation reports throughout the study as the response variable (i.e. persistent suicidal ideation, as previously defined), and sexual orientation (sexual minority vs heterosexual) as the predicting variable. Variables identified as potential confounders or effect modifiers, on a theoretical or statistical basis (i.e. due to differences between groups), were included in multivariable analysis. Planned sensitivity analyses controlled for country, an alternative definition of sexual minority status (i.e. excluding pupils responding “I don’t know”), and the SUPREME allocation group. The simple and multivariable regression analyses were repeated after stratifying the sample for sex (female and male). Odds ratios (OR; from 2x2 tables) and rate ratios (RR; from Poisson regression) were calculated together with their respective 95% confidence intervals (CI), and statistical significance was set at a p value < 0.05, two-tailed. The statistical analyses were performed with IBM SPSS Statistics for MacOS (version 25).

### Ethics statement

The SUPREME project was approved by the ethics committees of each national centre:

Estonia: Tallinna Meditsiiniuuringute Eetikakomitee (TMEK)
Hungary: Egészségügyi Tudományos Tanács Titkárság Tudományos es Kutatásetikai Bizottság (ETT TUKEB)
Italy: Comitato Bioetico Di Ateneo, Università Degli Studi Del Molise
Spain: Comité Ético de Investigación Clínica del Parc de Salut MAR
Sweden: Regionala etikprövningsnämnden i Stockholm, kansli vid Karolinska institutet
United Kingdom: Faculty (of Health, Social Care & Education) Research Ethics Panel, Anglia Ruskin University

Participation to the study was voluntary. Written consent was given by all participants and, depending on local regulations, by one or two caregivers.

## Results

**Fig 1:**
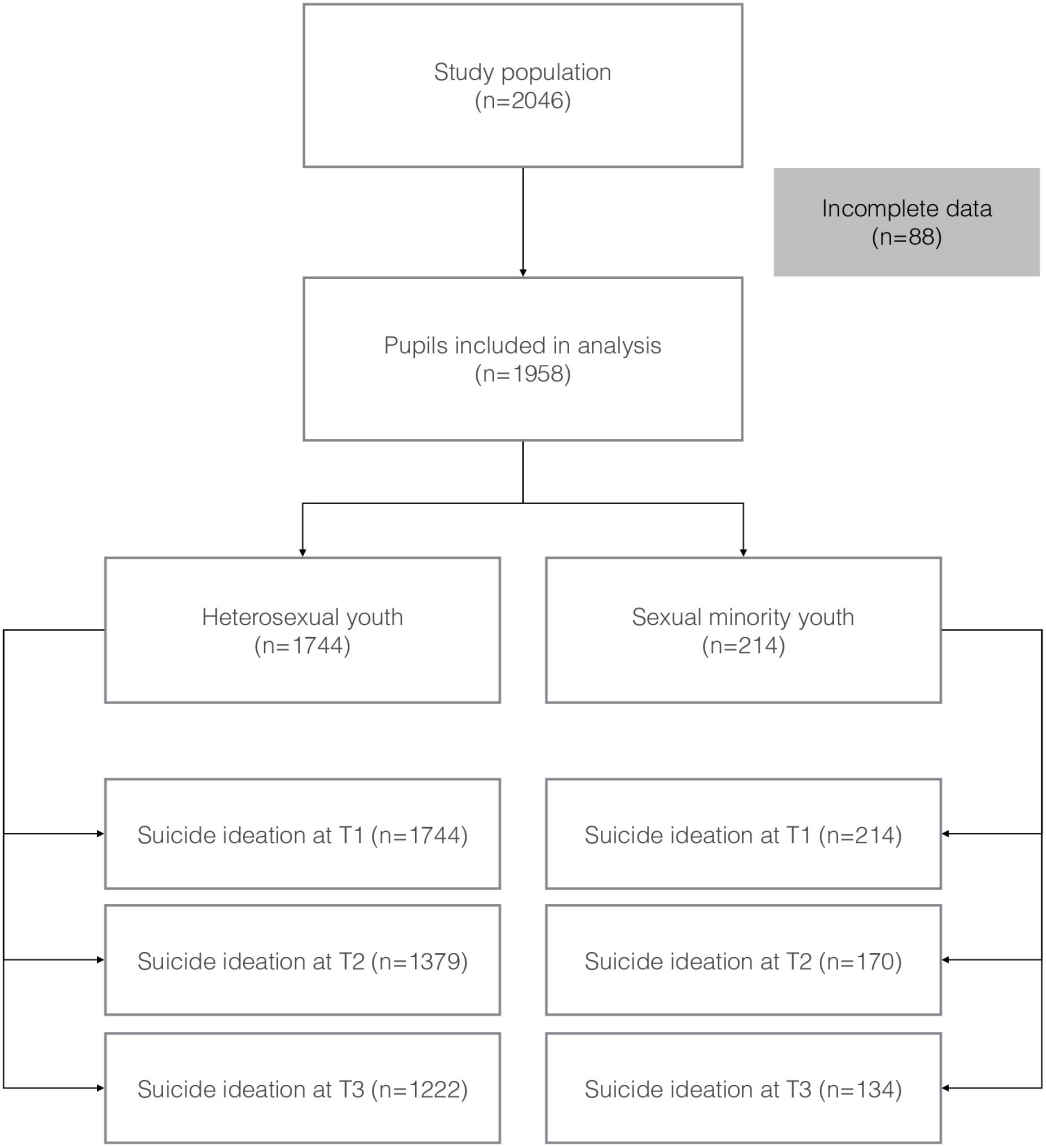
Study flowchart.

Complete data about sexual minority status, suicidal ideation, age and sex were available from a total of 1958 pupils (95.7% of the study population; median age 15; IQR 15–16; mean 15.35±1.00; females 56.8%) (Fig 1). Of them, 1744 pupils were categorized as heterosexual youth (HSY; 89.1%) and 214 as sexual minority youth (SMY; 10.9%). The two groups were homogeneous for age whereas significantly more females were among the SMY (74.3% vs 54.7% in the HSY group).

Other baseline characteristics of the sample and a comparison between the two groups are presented in Table 1. Exposure to bullying, school-related stress, and lower perceived economic status was significantly more common in the SMY group. However, similar rates of suboptimal family interaction were seen in HSY and SMY. The rates of very religious pupils or discording religiosity were not significantly different between the groups.

**Table 1:** Baseline characteristics of the study population in association with sexual orientation.

Variables	Respondents	Heterosexual youth	Sexual minority youth	
	*n (%)*	*n (%) or median (IQR)*	*n (%) or median (IQR)*	*p-value* ^a^
Total sample	1958 (100)	1744 (100)	214 (100)	
Age	1958 (100)	15 (15–16)	15 (15–17)	0.054
Sex (Female)	1958 (100)	954 (54.7)	159 (74.3)	<0.001
Substance abuse	1778 (90.8)	157 (9.9)	29 (14.8)	0.036
Bullying victimization	1936 (98.9)	866 (50.2)	140 (66.4)	<0.001
School-related stress	1939 (99.0)	547 (31.7)	89 (42.0)	0.003
Suboptimal family interaction	1919 (98.0)	205 (12.0)	35 (16.7)	0.050
Lower economic status	1938 (99.0)	174 (10.1)	35 (16.6)	0.004
High religiosity	1948 (99.5)	274 (15.8)	28 (13.1)	0.300
Discordant religiosity	1948 (99.5)	587 (33.9)	84 (39.3)	0.117
Previous suicide attempts	1926 (98.4)	104 (6.1)	31 (14.9)	<0.001
Suicidal ideation score	1941 (99.1)	1 (1–1.25)	1.25 (1–2)	<0.001

IQR: interquartile range.

^a^ Mann-Whitney U test (for continuous variables) or Chi-square test (for categorical variables).

At the baseline, SMY pupils had significantly higher PSS scores compared to their heterosexual peers (p<0.001; r 0,145). Previous suicide attempts were also more frequently reported by SMY pupils when compared to the HSY (OR 2.72; 95% confidence interval [CI] 1.77–4.18). The prevalence of serious suicidal ideation was significantly higher among the SMY compared to the HSY at T1 (OR 2.64, 95%CI 1.83–3.80), T2 (OR 2.50, 95%CI 1.56–3.99) and T3 (OR 2.72, 95%CI 1.54–4.80), respectively (Table 2).

**Table 2:** Sexual orientation and prevalence of serious suicidal ideation during the study.

Time-point ^a^	Sexual orientation	n (%) ^b^	OR (95% CI) ^c^	p-value ^d^
T1	Heterosexual youth	164 (9.4)		
	Sexual minority youth	46 (21.5)	2.64 (1.83–3.79)	< 0.001
T2	Heterosexual youth	93 (6.7)		
	Sexual minority youth	26 (15.3)	2.50 (1.56–3.99)	< 0.001
T3	Heterosexual youth	62 (5.1)		
	Sexual minority youth	17 (12.7)	2.72 (1.54–4.80)	< 0.001

^a^ Response rates are as follows: T1 100%; T2 79,1%; T3 69,3%.

^b^ Frequency and proportion of respondents reporting serious suicidal ideation at each time-point.

^c^ Odds ratios (OR) and 95% confidence intervals (CI) for serious suicidal ideation among sexual minority youth to heterosexual youth (reference category).

^d^ Chi-square test.

Almost 60% (1168 of 1958) of the pupils were available at all of the three study points and thus included in the Poisson regression analysis (Table 3). The rate of serious ideation reports was significantly higher among SMY when compared to HSY (RR 2.55, 95%CI 1.90–3.43, p < .001). The statistical significance was stable after adjustment for several covariates in multivariable analysis (aRR 1.73, 95%CI 1.23–2.48; Table 3). The same applied to the planned sensitivity analyses controlling for country, SUPREME allocation group and for an alternative definition of the exposure variable. The sex-stratified analysis revealed a significant association between SMY status and persistent suicidal ideation for both females and males. The estimates for male pupils were notably higher than those for female pupils, in both simple and multivariable analysis, although confidence intervals were overlapping (Table 3).

**Table 3:** Sexual orientation and persistent serious suicidal ideation during the study.

	Total sample	Females	Males
*Simple regression* ^a^	*RR (95%CI)*	*RR (95%CI)*	*RR (95%CI)*
Sexual orientation (SMY)	2.55 (1.90–3.43) ***	2.05 (1.46–2.88) ***	3.37 (1.81–6.26) ***
*Multivariable regression* ^b^	*aRR (95%CI)*	*aRR (95%CI)*	*aRR (95%CI)*
Sexual orientation (SMY)	1.73 (1.23–2.48) **	1.51 (1.01–2.24) *	3.84 (1.94–7.59) ***
Sex (Female)	1.47 (1.08–2.02) *	-	-
Age	0.96 (0.83–1.11) °	0.87 (0.73–1.04) °	1.25 (0.94–1.67) °
Substance abuse	1.87 (1.30–2.69) **	2.16 (1.41–3.33) ***	1.29 (0.64–2.61) °
Bullying victimization	3.23 (2.28–4.57) ***	3.97 (2.57–6.14) ***	2.03 (1.12–3.67) *
School-related stress	1.76 (1.33–2.34) ***	1.79 (1.28–2.49) **	1.60 (0.89–2.86) °
Suboptimal family interaction	2.77 (2.02–3.79) ***	2.35 (1.62–3.41) ***	4.40 (2.42–8.01) ***
Lower economic status	1.98 (1.41–2.79) ***	1.81 (1.20–2.74) **	3.13 (1.68–5.86) ***
Discordant religiosity	1.38 (1.05–1.82) *	1.37 (0.98–1.90) °	1.34 (0.78–2.29) °

Poisson regression derived rate ratios (simple [RR] and adjusted [aRR]) of serious suicidal ideation reported during the study in relation to sexual orientation and other variables. P-values are as follows:

° p ≥0.05;

* p <0.05;

** p <0.01;

*** p <0.001.

^a^ N = 1168 (females 55.7%; SMY 9.9%).

^b^ N = 1026 (females 55.3%; SMY 10.1%).

## Discussion

### Findings

This longitudinal study evaluated the association between sexual orientation and suicidal ideation in a multinational sample of two thousand European adolescents. Pupils belonging to a sexual minority were found to have significantly higher scores of suicide ideation as well as a higher prevalence of serious suicidal ideation and of previous suicide attempts in comparison to their heterosexual peers. In regression analyses, non-heterosexual orientation significantly predicted persistent suicide ideation. Compared to their heterosexual peers, SMY had 2.55 (95%CI 1.90–3.43) times more reports of serious suicide ideation during the study, a statistically significant difference (p< .001). A significant association was found even after adjustments for sex, age and several other variables in multivariable analysis (RR 1.73; 95%CI 1.23–2.48). In sex-stratified analyses, significant differences were respectively found among female and male pupils, both in univariable and multivariable analysis. Interestingly, the association between SMY status and the counts of severe suicidal ideation was particularly strong among male pupils (aRR 3.84; 95%CI 1.94–7.59).

### Strengths and limitations

The multinational population in this study consisted of adolescent pupils from 27 schools which were randomly selected in six European countries, thus representing a large geographic area. The SUPREME project followed a rigorous protocol and was centrally coordinated in order to ensure homogeneous practice across the study sites. The main outcome was measured prospectively over time, rather than cross-sectionally as in most available studies. Several factors could be analyzed and adjusted for in the multivariable analysis. Furthermore, sex stratification allowed for the evaluation of differences between females and males, who are known to be different in terms of suicidal behavior and social interaction.

A number of limitations need to be considered. The exposure and outcome variables were obtained via self-report, and not all the selected cohort was available for analysis at all study points. However, a high percentage of participants were available at the two post-baseline time points respectively (T2, 80%; T3, 70%), and 60% were available at all the three time points. The exposure variable in the study only addressed sexual orientation, whereas no information was available about gender identity, another potential correlate of minority stressors. Similarly, the questionnaire did not address sexual behavior, which is related to adolescent mental health [3,5] and may be a predictor of suicidality among sexual minority youth [18].

### Interpretation and implications

Adolescence is a period of intense development as well as of exploration and social changes. As a result, adolescents are particularly vulnerable and many of them experience emotional and behavioral problems [2,5]. Suicide is one of the top three leading causes of death among adolescents globally, and it is therefore a priority target for preventive efforts in adolescent health [1].

Potential sources of stress and mental health risk during adolescence are related to physiological developmental milestones such as sexual attraction, identity exploration and initiation [3–5]. In the present study, we found a higher prevalence of suicidal behavior among adolescents belonging to a sexual minority, i.e. those who do not identify themselves as heterosexual, when compared to their heterosexual peers. These findings are in line with previous observations [7–9] and may be interpreted within the so-called minority stress model [10]. The latter explains the excess of mental ill-health among individuals belonging to a sexual minority as the product of stressors such as discrimination, victimization and stigma.

Because of the social dimension of such minority stressors, mental health outcomes in research studies are likely to depend on the context from which the samples originate. Our study is one of the few so far existing to confirm the association between sexual minority status and suicidality in a European multinational sample, whereas most of the published observations to date are based on cross-sectional samples of North-American adolescents [7–9]. These findings are therefore an important contribution because, according to a recent meta-analysis on attempted suicide among sexual minority youth, the origin of the samples is a source of statistical heterogeneity [9]. In the same meta-analysis, no heterogeneity was found between the only two single-nation studies from Europe (from Ireland [19] and Switzerland [20]) [9]. Interestingly, the stability of our findings after controlling for country also suggests that differences among countries at European level may not be significant. However, SUPREME’s sampling strategy did not account for sexual orientation, whereas serious suicidal ideation is a low-prevalence outcome. The resulting national samples may therefore not allow to reliably test or interpret variations in the association between sexual minority status and suicidality among the participating countries.

Suicidal ideation is not infrequent among adolescents regardless of their sexual orientation [2,3]. In fact, the difference in suicidal ideation scores between sexual minority and heterosexual youth in our study, although significant, was relatively small. However, sexual minority status was strongly associated with more serious manifestations of suicidality, such as suicide attempts or planning. It was therefore important to include the latter as outcomes in the study because previous research has shown that the disparity between heterosexual and sexual minority youth is larger when more severe suicidal behavior is considered [7]. In other words, SMY do not just have higher rates of suicidality but the suicidal behavior among SMY is more serious than that observed among heterosexual youth.

In addition, the analysis of longitudinal data in the present study shows that SMY have more persistent suicidal ideation compared to their heterosexual peers, independently from a number of potentially related stressors. This suggests a chronic exposure to minority stressors which results in the additive effect of distal and proximal factors on mental outcomes [10]. As a matter of fact, life events and social interaction are often source of distal stressors (i.e. external or objective events and conditions) during adolescence [2,5]. However, a chronic exposure to such distal minority stressors eventually leads to proximal, personal, processes such as expectation of rejection, concealment or internalized stigma [10].

Our study finally suggests that SMY status is a stronger predictor of suicide ideation among male, rather than female, adolescents although the relative confidence intervals in our stratified analysis were overlapping. It should be noted that males, despite higher suicide rates, commonly report less suicide ideation than females [2]. Besides, sex did not moderate the association between SMY status and suicidality in a previous meta-analysis [7]. A difference between females and males is however plausible, because of gender norms which may result in an excess of distal (e.g. bullying) but also proximal (e.g. internalized homophobia) stressors among males.

A major implication of these findings is the need to recognize sexual minority youth as a high-risk group for suicide, even independently from objective factors such as victimization or substance abuse. Interventions grounded on knowledge about risk factors in the general adolescent population may overlook the disparities related to sexual orientation thus be less effective in preventing suicide in sexual minority groups. Preventive strategies should be adapted for SMY and consider the management of context vulnerabilities and related distal stressors, before the establishment of proximal stressors. The important protective role of social interactions and economic factors should be considered, as well as the possibility that different interventions are likely to be needed for female and male SMY.

In addition, these findings suggest several implications for further research. The differential effectiveness of known suicide preventive interventions among sexual minority and heterosexual youth should be evaluated. Possibly, specific interventions for SMY could be integrated into existing strategies and tested. As psychological distress and suicidality during adolescence are likely to continue during young adulthood and beyond, further longitudinal studies would help assessing specific trajectories and the potential effect of secondary preventive measures. Since SMY may be more exposed than heterosexuals to risk behaviors, such as substance abuse and risky sexuality [21–23], research should consider physical health outcomes aside the mental health ones.

## Conclusions

Among European adolescents, a non-heterosexual orientation is strongly associated with serious and persisting suicidality. These findings suggest that there is a need to develop primary and secondary preventive measures for sexual minority youth. At the same time, existing suicide-preventive strategies need to account for sexual orientation as well as for the differences between female and male sexual minority youth. Context-targeting interventions should be implemented, with specific focus on social interactions among peers and within families, as well as on economic factors.
